# TSST-home – A pilot study investigating a home version of the ‘Trier Social Stress Test’

**DOI:** 10.1016/j.cpnec.2026.100369

**Published:** 2026-07-24

**Authors:** Nadine Skoluda, Nida Ali, Urs M. Nater

**Affiliations:** a Faculty of Psychology, University of Vienna, Vienna, Austria; b Department of Psychology, Queen's University, Kingston, Canada

**Keywords:** Trier social stress test, TSST, Cortisol, Alpha-amylase, Heart rate, Home setting

## Abstract

The Trier Social Stress Test (TSST) is a well-established laboratory stress-inducing paradigm. However, its implementation requires a research environment with dedicated personnel (usually at a research institution). When participants cannot travel to a research institution (due to age, mobility, or geographical restrictions), a home version of the TSST is needed. We have developed the TSST-home, which shares the essential characteristics with the laboratory version but is implemented at the participant's home. In this pilot study, we aimed to investigate the feasibility of the TSST-home and its ability to induce stress responses in comparison to a non-stressful control condition.

Seventeen healthy men (mean age and range: 24 years; 18-30 years) were exposed, in randomized order, to the TSST-home and the placebo-TSST-home (control condition) in their respective homes. During each session we assessed perceived stress, salivary cortisol, salivary alpha-amylase, heart rate to account for the multifaceted nature of acute stress responses.

Conservative and liberal cortisol responder rates (≥2.5 and ≥ 1.5 nmol/l increase) in the TSST-home condition were 70% and 82%, respectively. The TSST-home and placebo-TSST-home differed significantly in their trajectories of stress markers with stronger stress-induced responses across all stress measures in the TSST-home condition.

Findings suggest that the TSST-home elicits reliable and valid acute stress responses across different stress markers comparable to those found in the original laboratory-based TSST. This tool can be used to investigate stress responses of participants who cannot otherwise participate in laboratory-based studies at research institutions.

## Highlights

• TSST-home is an adapted stress paradigm conducted in the participant's home.
• Cortisol, alpha-amylase, heart rate, and perceived stress were assessed.
• All stress markers increased in response to the TSST-home.
• TSST-home elicits stronger multi-faceted stress responses than a placebo version.
• TSST-home is a novel stress tool for field and epidemiological studies.

## 1 Introduction

The Trier Social Stress Test (TSST) [1] is a well-established laboratory paradigm aimed to elicit reliable acute psychobiological stress responses [2]. Over the decades, researchers have developed various modified versions that are adapted to participants' characteristics (e.g., young age [3,4]) or the modern digital world (e.g., virtual-reality [5], or online-based TSST [6])). The laboratory setting, usually located at a research institute, is associated with challenges, such as travel burden for participants, and environmental and spatial requirements for researchers. For potential participants who are unable to travel to a research institution (due to age, mobility, or geographical restrictions), a home version of the TSST may be a solution. There are online versions of the TSST [[6], [7], [8]] which allow individuals to participate from home, however, they require certain technical conditions (e.g., computer device including camera, stable internet connection), and lack ‘real’ in-person interactions, reportedly resulting in lower cortisol responses than the original laboratory-based TSST [7]. We have developed the TSST-home, which overcomes these limitations and shares the essential characteristics with the laboratory version. The aim of the current within-subject, crossover, single-blind study was to investigate the feasibility and ability of the TSST-home to elicit stress responses across multiple systems (i.e. biological and psychological). We hypothesize that compared to the placebo-TSST-home, the TSST-home will elicit significant acute stress responses across the most-commonly assessed stress markers (i.e., perceived stress, salivary cortisol, salivary alpha-amylase, heart rate).

## 2 Methods

### 2.1 Participants

Prospective participants were recruited via flyers (e.g., notice boards of local universities) and social media (Facebook) and screened using a telephone-based interview. For this pilot study, we studied a relatively homogenous sample of healthy, young men to control for possible confounding effects on the stress measures. The inclusion criteria were the following: a) male biological sex/gender b) young adults (18-35 years), c) BMI 18-25 kg/cm^2^, d) fluency in German, and e) living in Vienna, with at least two separate rooms for conducting the study. We excluded participants who reported a) regular smoking (≥5 cigarettes/week), b) drug/medication use, c) mental/somatic disorders, d) (night) shift work, and e) previous experience with the TSST. Participants were compensated 80 € for their study participation. A summary of the characteristics of the participants’ home settings and their preference for study location (university vs. home) is provided in Appendix C.

Using G*Power [9] for the priori sample size estimation (expected medium effect size of 0.25, power of 0.90, p value of 0.05, two conditions, eight time points), at least 24 participants were required. Data collection occurred between February 2020 and June 2023. Given that this timeframe overlapped with the Covid-19 pandemic (Covid-19 measures were implemented accordingly, see Appendix B) and associated repeated lockdowns and governmental restrictions, our final study sample was smaller than originally planned. For this pilot study, we report results for 17 male participants (mean age ± standard deviation, range: 23.59 ± 2.92, 18-30 years; BMI: 22.35 ± 1.41, 19.10-24.78 kg/m^2^).

### 2.2 Procedure

A detailed illustration of the procedure is provided in Appendix A. After participants completed an online home survey (Unipark, EFS Survey software, Tivian XI GmbH, Munich, Germany) assessing their characteristics (e.g., sociodemographic factors), they completed the TSST-home and placebo-TSST-home in randomized order at home. Participants were blinded to the order of the condition, and thus, did know which task serves as a stress task. Seven participants completed the TSST-home first; ten completed the placebo-TSST-home first. Sessions were held on two separate days (both sessions either on weekdays or weekends within participants), with 5-8 days between sessions. Sessions were scheduled in the afternoon (2.00-4.00 p.m.) to account for the diurnal rhythm of stress markers. Participants gave informed consent. This study was performed in accordance with the Declaration of Helsinki and was approved by the Ethics Committee of the University of Vienna.

### 2.3 Sessions: TSST-home and placebo-TSST-home

The experimenter arrived at participants' homes shortly before 2.00 p.m. During the first session, participants provided written informed consent and showed their two rooms (for testing) to the experimenter, who decided which room would serve as the ‘participant room’ (room A), in which participants would spend most of the time during the session, and the ‘TSST room’ (room B). Participants were instructed to self-collect saliva samples (for the later assessment of cortisol and alpha-amylase) and to use time trackers on a sensor device (for the assessment of heart rate).

For the assessment of the baseline measurements, there was a 30-min acclimation period in room A during which the sensor device was applied, and the participants completed questionnaires and read magazines (pre-screened for non-stimulating content). During this period, the TSST committee (1 male, 1 female) arrived at the apartment, avoiding any encounter with participants, and set up testing equipment in room B. At the end of the acclimation period, the first baseline stress measurements were conducted (−10 min relative to the (placebo-)TSST). Specifically, participants provided the first saliva samples, set a time marker on the sensor device, and completed momentary psychological stress measures. The experimenter then accompanied participants to room B, where they were either exposed to the TSST-home or the placebo-TSST-home, which was adapted from the previously published ‘placebo TSST’ [10]. For this, the experimenter gave instructions to the participants to prepare a speech (TSST: mock job interview in front of the present committee, the participants were told that they would be video-recorded; placebo-TSST-home: speech about a pleasant topic of their own choosing (such as a favorite movie, novel)). After a 3-min preparation period, the second measurements were conducted (pre-task, −1 min), and the experimenter left the room. Following the TSST protocol, the participants gave a 5-min speech and solved a 5-min mental arithmetic task (serial subtraction: 2043-17) in a standing posture in front of the TSST committee. The committee comprised two judges, dressed in white laboratory coats, sitting behind a table, offering brief, neutral feedback. In the placebo-TSST-home condition, participants delivered a speech while standing alone in room B (without any TSST equipment such as video-camera and microphone). After 5 min, the experimenter entered, gave instructions for the arithmetic task (serial addition: 100 + 5), and left again. After completing the tasks, the experimenter re-entered, and the third measurements (post-task, +1 min) were conducted. Afterwards, the experimenter and participants returned to room A, where the participants completed questionnaires and read magazines during a 1-h recovery period. During the recovery period, another five measurements (+10 min, +20 min, +30 min, +45 min, +60 min relative to the (placebo-)TSST) were obtained. At the end of the session, participants were debriefed by the experimenter.

### 2.4 Stress response measures

In this report, we focus on measures for assessing multi-system stress responses. Salivary samples were collected using SaliCap devices (IBL-Tecan group, Hamburg, Germany) during the sessions (−10, −1, +1, +10, +20, +30, +45, +60 min relative to the (placebo-)TSST). Participants were instructed to collect saliva for 2 min and transfer it into a tube using a straw. *Cortisol* concentrations (sCort) were analyzed using a commercially available luminescence immunoassay (IBL-Tecan group, Hamburg, Germany). *Alpha-amylase* activity (sAA) was determined by an enzyme kinetic method using reagents by DiaSys Diagnostic Systems (Holzheim, Germany) [11]. Intra- and inter-assay coefficients of variation were below 10% for both measures. Using sensor devices (movisens GmbH, Karlsruhe, Germany), *heart rate* (HR) was recorded during the sessions. To assess the course of HR over the sessions, averaged 5-min intervals were calculated for baseline (last 5 min of the acclimation period), speech preparation and anticipation, speech, arithmetic task, post task (0-5 min after task), recovery I (5-10 min after task), recovery II (20-25 min after task), recovery III (30-35 min after task), recovery IV (45-50 min after task), and recovery V (55-60 min after task). Simultaneously with the saliva sampling timepoints, *perceived stress* was assessed by using a visual analogue scale (VAS, 100 mm), asking “How stressed do you feel?”, with ‘0’ not at all and ‘100’ very.

### 2.5 Statistical analyses

#### 2.5.1 Missing data

One saliva sample (timepoint #4, +10 min after the placebo-TSST) from one participant had insufficient volume for analysis. One participant provided no data on the VAS (timepoint #2, −1 min before TSST).

#### 2.5.2 Comparison in stress response

We report cortisol responder rates to the (placebo-)TSST-home according to the conservative and liberal classification criteria, with ≥2.5 nmol/l [1] and ≥1.5 nmol/l increase from baseline to peak [12] indicating stress responses, respectively.

Mixed-effects multilevel models (MELM [13]) were computed to examine the relationship between condition and repeated log-transformed measures of biological (sCort, sAA, HR) and psychological (VAS) stress response measures. Sampling times (time 1 = 0, time 2 = 1, etc.), condition (placebo-TSST-home = 0, TSST-home = 1), and time × condition interaction were entered as fixed effects. Participants' intercepts were added as a random effect. Condition order (1 = 0, 2 = 1) was included as a covariate. MELMs were computed in a stepwise manner. For each stress marker, we first computed a covariate-only model. Model 2 added time as a predictor. In model 3, in addition to time, condition was added as a predictor. The final model included the interaction term for time and condition. Likelihood ratio tests were conducted to compare the final model with the identical model except for the fixed effect in question. For ease of reading, here we report only the results of the best-performing model. All analyses were computed using R Core Team (2025) version 4.5.1. The lme4 package was used for MELM analyses [14]. Significant effects were decomposed using the emmeans package [15].

In Appendix D, we additionally report results the MELM statistics using non-transformed data as well as the findings of the Bayesian statistics, another statistical approach to investigate the effectiveness of the TSST-home.

## 3 Results

**Fig. 1:**
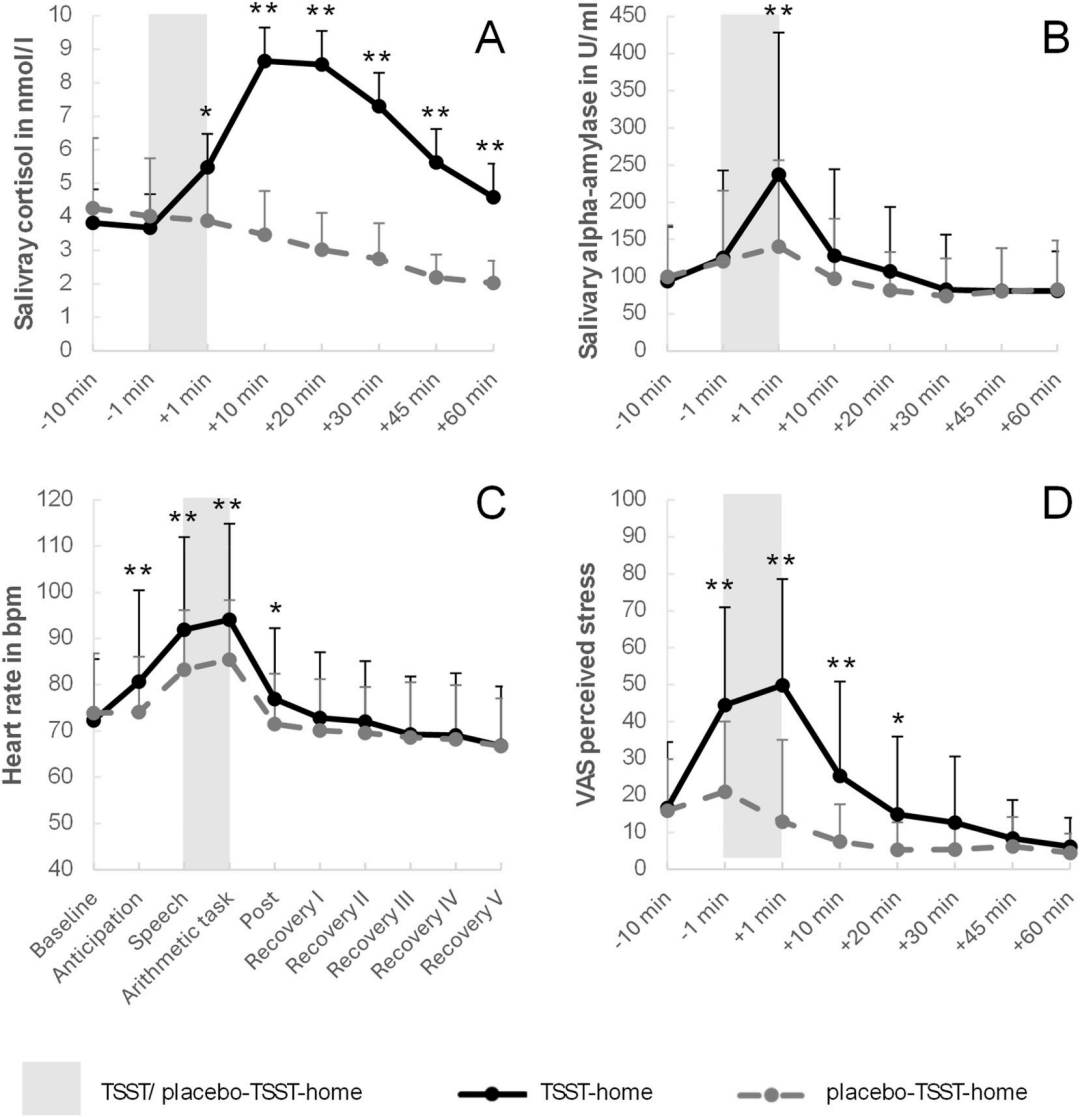
Trajectories of stress responses: A) salivary cortisol, B) salivary alpha-amylase, C) heart rate, D) VAS perceived stress. Note, error bars correspond with standard deviations. *p < 0.05; **p < 0.01 Salivary samples and VAS perceived stress were obtained at the following timepoints: −10, −1, +1, +10, +20, +30, +45, and +60 min relative to the task. For heart rate, averaged 5-min intervals were calculated for baseline (at the end of the acclimation period, corresponding 10-15 min before task), speech preparation and anticipation (corresponding 5 min before speech), speech, arithmetic task, post task (0-5 min after task), recovery I (5-10 min after task), recovery II (20-25 min after task), recovery III (30-35 min after task), recovery IV (45-50 min after task), and recovery V (55-60 min after task).

Using conservative (≥2.5 nmol/l) and liberal (≥1.5 nmol/l) classification criteria, 12 (70%) and 14 (82%) participants can be classified as cortisol responders to the TSST-Home, respectively. In the placebo-TSST-home condition, no participants met the conservative criteria for cortisol responders, and one participant met the liberal criteria for cortisol responders (exposed to the placebo condition first). Fig. 1 illustrates the trajectories of stress markers. The MELMs revealed significant time × condition interactions for sCort: *F*(7, 239) = 16.73, *p* < 0.001; sAA: *F*(7, 239) = 2.98, *p* < 0.01; HR: *F*(9, 304) = 2.47, *p* < 0.001; VAS: *F*(7, 239.02) = 2.71, *p* < 0.05. Simple effects of condition for each time point were probed using estimated marginal means with Tukey-adjusted pairwise comparisons. Results revealed that, as predicted, the TSST-home induced stronger sCort responses to stress than the placebo-TSST-home, corresponding with all timepoints associated with stress response and recovery (timepoints 3-8; all *t*s > 2.82; ps ≤ 0.01). Likewise, for sAA, HR, and VAS, compared to the placebo-TSST-home, stress induction via the TSST-home was associated with stronger reactive increases, corresponding with all timepoints associated with stress response: sAA (timepoint 3; *t* = 4.43; *p* ≤ 0.001); HR (timepoints 2, 3, 4, 5; all *t*s > 2.6; *p*s ≤ 0.001); VAS (timepoints 2, 3, 4, 5; all *t*s > 2.2; *p*s < 0.05).

## 4 Discussion

This study proves that it is (logistically) feasible to implement the TSST at the participants’ home, even during epidemiological crises such as the Covid-19 pandemic. The findings of this pilot study further suggest that compared to the placebo-TSST-home, the TSST-home successfully elicits stress responses across multi-stress systems (sCort, sAA, HR, and VAS).

In line with studies using laboratory [2] and online versions of the TSST [[6], [7], [8]], we found increases in sCort, sAA, HR, and VAS as a response to the in-person TSST-home. The conservative cortisol responder rate (≥2.5 nmol/l increase) was 70% in our study which corresponds to those reported for the ‘original’ laboratory-based TSST [1], indicating comparable cortisol responses between the laboratory-based TSST and the TSST-home. Using a more liberal classification criterion (≥1.5 nmol/l increase), we found a higher cortisol responder rate (82%) in our study than those reported for online-based TSSTs (33% [7], 64% [8]). It is conceivable that the lack of an in-person interaction in the online-based TSST may mitigate the cortisol stress response, with individuals likely distancing themselves from social-evaluative threat presented only onscreen. According to the liberal criteria, one participant responded to the placebo-TSST. The novelty und unpredictability of the situation might have caused measurable stress responses in this individual as the placebo-TSST was randomly scheduled as the first condition.

Although we could show significant responses to the TSST-home in this pilot study, the small sample size is a limitation of this study. Although more robust Bayesian statistics (see suppl. D.3) generally confirmed our findings, the results need to be corroborated in a larger sample. Moreover, our study included exclusively healthy men aged 18-30 years. Thus, these findings may not generalize to patient samples, other genders and age groups. Future studies should replicate the study using a representative and diverse sample. Our study allows us to evaluate stress responses to the TSST-home (in comparison to a placebo-version). However, the comparison between laboratory-based and home-based TSST is limited to generally reported cortisol responder rates. Thus, future studies should include a laboratory-based TSST condition to directly compare stress responses between laboratory and home settings. Moreover, a systematic review is needed to compare the various TSST paradigms (laboratory-based classical [1], virtual-reality [5], online-based [[6], [7], [8]], and home-based TSST) which may help researchers to make informed decisions about which stress induction is best-fitting to their study design (a brief comparison is provided in Appendix E).

In conclusion, the TSST-home provides a feasible stress-inducing tool that can be implemented at individuals’ homes, increasing the ecological validity of this stress test compared to laboratory-based TSSTs. It can be used in field studies and epidemiological studies investigating population-based samples who cannot travel to a research institution.

## Declaration of generative AI and AI-assisted technologies in the manuscript preparation process

All authors declare that they did not use any generative AI and AI-assisted technologies in the manuscript preparation process.

## CRediT authorship contribution statement

**Nadine Skoluda:** Conceptualization, Data curation, Investigation, Methodology, Project administration, Supervision, Visualization, Writing – original draft, Writing – review & editing. **Nida Ali:** Conceptualization, Formal analysis, Methodology, Visualization, Writing – original draft, Writing – review & editing. **Urs M. Nater:** Conceptualization, Methodology, Resources, Supervision, Writing – original draft, Writing – review & editing.

## Declaration of competing interest

The authors **NA, NS, UMN** declare that they have no known competing financial interests or personal relationships that could have appeared to influence the work reported in this paper.

## Footnotes

Appendix A Supplementary data to this article can be found online at https://doi.org/10.1016/j.cpnec.2026.100369.
