# Supplementary material for: TSST-home – A pilot study investigating a home version of the ‘Trier Social Stress Test’
Source: Compr Psychoneuroendocrinol. 2026 Jul 24;27:100369. doi: 10.1016/j.cpnec.2026.100369 (PMC13451864; doi:10.1016/j.cpnec.2026.100369)
Supplement: Supplementary file 1 [file mmc1.docx]

**Appendix A.1: Overall Procedure**

|  | **Recruitment** |
| --- | --- |
|  | - Flyer (e.g., notice boards at local universities) |
|  | - Social media (e.g., Facebook) |
|  | 🡇 |
|  | **Screening: telephone-based interview** |
|  | - Inclusion and exclusion criteria |
|  | - Schedule appointments (2 home sessions) |
|  | 🡇 |
|  | **Online-based home survey** |
|  | - Questionnaires battery (e.g., sociodemographic, mental health, childhood trauma, perceived stress reactivity, stress coping, resilience, emotion regulation, self-esteem, self-efficiency, trait anxiety, trait anger expression, home characteristics) |
|  | 🡇 |
|  | **Two home sessions** |
|  | - TSST-home and placebo-TSST-home in randomized order, 5-8 days apart (both sessions either on weekdays or weekend days within the same participants): repeated assessment of stress measures |
|  | - Questionnaires during baseline and recovery period (stress, fatigue, depression, Big Five personality traits) |

Figure A.1: Overall procedure of the study

Note, data collected from questionnaires are used for sample description, and in a larger sample, these data allow the investigation of possible factors that are known to affect acute stress responses.

**Appendix A.2: Procedure of home sessions**

Figure A.2: Procedure of home sessions (TSST-home, placebo-TSST-home).

**Appendix A.3 Standardization of the TSST-home**

(Pre-)Selection of the rooms

During the telephone-based interview, participants were asked about their home conditions to evaluate whether they fulfill the inclusion criteria (see also A3.1 interview script). At the first visit, the experimenter selected which rooms served as “participant room” (room A) and “TSST room” (room B), based on the information provided in the telephone-based interview and experimenter’s own impression. The two rooms need to be separated from each other (i.e., rooms need to be closed by a door). Furthermore, the two rooms need to provide the required space, and if possible, the required furniture (please see below).

Set-up of the participant room:

A table and chair for the participant room were required so that the participants sit at the table where they complete questionnaires and read magazines. An additional chair was used by the experimenter. It was positioned in a calling distance, allowing the participant some “privacy” during the baseline and recovery period.

Set-up of the TSST room:

The TSST committee brought all the required TSST equipment using a trolley bag (W x H x D: 85 x 43 x 44 cm; including white lab coats, video camera, tripod, microphone, writing materials, white-colored tablecloth). During the Covid-19 pandemic, a bag with the mobile walls was additionally transported to participants’ house.

If available, tables and chairs in participants’ home were used for the TSST. If no suitable furniture was available, the research team brought a white-colored collapsible camping table (length x width x height: 183 x 76 x 74 cm) and two black-colored height-adjustable folding stools to participants’ home. Additionally, a white-colored tablecloth was provided. A microphone was placed on the table close to the participant. A video camera placed on a tripod was set directly behind and centered between the TSST committee.

For reasons of standardization, the distance between participants and the table, where the TSST committee was seated, measured 1.50 meters (± 10 cm). In a pre-test among research team members, we noticed that this distance is ideal to create a professional distance (neither too close to intrude into individual’s personal space, nor too far to create dis-involvement). Participants stand on a marked “x” (easily removable, beige-colored crepe paper) on the ground.

In the placebo-TSST participant also stand on the marked “x” to increase comparability between the two conditions. There was no additional equipment in the room.

TSST committee:

The stress committee consisted of one male and one female member who wore white lab coats and neutral footwear (e.g., black or white socks). Similarly to the laboratory-based TSST, previous encounters between participants and the TSST committee were avoided. Thus, participants met the TSST committee not until when they were introduced to the TSST.

Experimenter:

The experimenter had a normal-sized back bag (W x H x D: 34,3 x 46,6 x 23 cm) that included all the necessary study material (e.g., informed consent, questionnaires, saliva sampling material, sensor device for the assessment of heart rate).

**A3.1 Telephone-based interview: questions regarding living conditions**

For participation in the study, your home must meet certain requirements: We need two separated rooms which can be used for about two hours without any disturbances. Thus, I have some questions about your living conditions.

(German: Für die Studie müssen passende räumliche Gegebenheiten vorliegen. Wir benötigen dafür zwei voneinander getrennte Räume, die wir während des Versuchs, der ungefähr 2 Stunden dauert, ungestört nutzen können. Ich hätte daher noch einige Fragen zu Ihren Wohnverhältnissen.)

- How long do you live in your current apartment? (German: Wie lange wohnen Sie schon in Ihrer aktuellen Wohnung?) __________________________
- For logistic reasons, we can include only participants from Vienna and within a certain radius. Where do you live? (German: Aus organisatorischen Gründen können wir die Studie aktuell nur in Wien und in einem gewissen Umkreis durchführen. Wo sind Sie wohnhaft?) ____________________________________

*(Please note only city and district; if included, ask for postal address; German: bitte nur Ort und Bezirk angegeben; bei Einschluss nach genauer Adresse fragen)*

- Would you please describe briefly your apartment in your own words? How many rooms does your apartment have? How is the apartment structured? How big are the rooms? (German: Können Sie mir Ihre Wohnung kurz in Ihren eigenen Worten beschreiben? Aus wie vielen Räumen besteht sie, wie ist sie aufgebaut, und wie groß sind die Räume in etwa?)
- Covid-19-specific question: Is it possible to keep the minimum distance of 1 meter between individuals? It is possible to open the window for ventilation (German: Kann der Mindestabstand von 1 m zwischen den Personen eingehalten werden? Ist es möglich, die Fenster zum Lüften zu öffnen?)
- How many tables and chairs are available in the respective rooms? How big are the tables? (German: Wie viele Tische und Stühle stehen in den jeweiligen Räumen zur Verfügung? Wie groß sind die Tische in etwa?)
- Are you living on your own? Are there other individials living in your apartment, such as your parents, roommates, partner (German: Wohnen Sie allein oder leben in Ihrer Wohnung noch andere Personen wie Eltern, Mitbewohner*innen oder Ihre Partnerin bzw. Ihr Partner?)
- Do you have pets? (German: Haben Sie Haustiere?)
- Is it possible that we can conduct the study at your home without any disturbance? This particularly means that the individuals who live with you know about the study, give their consent, and do not spend time in any of the rooms we occupy during the time we conduct the study. *If necessary, please ask whether there is any construction work in or at the building.* (German: Haben wir in Ihrer Wohnung die Möglichkeit, die Testung ungestört durchzuführen? Dazu gehört vor allem, dass Ihre Mitbewohner*innen über die Studienteilnahme informiert und einverstanden sind und sich zum Zeitpunkt der Testung nicht in den Räumen aufhalten, die wir für die Studie verwenden. *Gegebenenfalls auch nachfragen:* Bauarbeiten im Haus?)

Additional space for notes or sketches (if applicable, use the back side of the paper):

(German: Freies Feld für Notizen/Skizzen zur Wohnsituation (ggf. Rückseite verwenden):)

If roommate, an informed consent is neccessary (German: Wenn Mitbewohner*innen, dann Einverständnis erfragen):

It is important that all individuals living in your apartment give their consent to conduct the study at your apartment. Have you already talked to your roommates about this study? Did they give their consent? (German: Es ist außerdem wichtig, dass Ihre Mitbewohner*innen damit einverstanden sind, die Studie bei Ihnen zu Hause durchzuführen. Haben Sie bereits mit Ihren Mitbewohner*innen über die Studie gesprochen? Ist/ Sind er/ sie damit einverstanden?)

□ No, he or she does not give his/ her consent [exclusion] (German: nein, er/ sie ist/ sind nicht damit einverstanden [Ausschluss])

□ Yes, he or she gives his/ her consent [inclusion] (German: ja, Mitbewohner*innen wissen Bescheid und sind einverstanden [Einschluss])

□ I do not know, I need to talk to my roommate(s) 🡪 please getting back to us in this regard via e-mail (German: weiß nicht; ich muss noch meine Mitbewohner*Innen fragen 🡪 bitte per Email Rückmeldung)

**Appendix B.1: Covid-19 measures**

Home visits were conducted in accordance with the governmental and university Covid-19 guidelines (University of Vienna, Austria) at the respective time of data collection.

Briefly, at time of a lockdown or contact restrictions (e.g., person-contact of not more than two different households at a time) data collection was paused.

At home sessions, the study team followed *general hygiene rules* such as washing hands at arrival and disinfecting hands if applicable. The experimenter wore *nitrile gloves* when handling saliva samples. All materials (e.g. biro, container for saliva samples) were *disinfected* after each home visit. In general, all involved individuals (experimenter, participants, members of the TSST committee) were instructed to keep *physical distance* of at least 1-2 meters. *Mobile walls* (plexiglass) were used to protect each member of the TSST committee and the participants. Except for the period during which the (placebo-)TSST took place, all involved individuals had to wear (FFP2) *face masks*. The experimenter *opened the windows* on a regular basis to air the room and reduce possible viral load. All involved individuals completed a questionnaire asking about Covid-19 symptoms within last 14 days (fever, repeated dry coughing, sore throat, shortness of breath, change of sense of smell or taste), and their temperature at the forehead was measured at the beginning of the study (see questionnaire below, Appendix B.2). Depending on the guidelines, all involved individuals further needed to provide a *valid negative test* (PCR test or rapid antigen self-test) and were asked about risk factors (corona risk areas, quarantine).

**Appendix B.2: Covid-19 questionnaire**

Have you had **fever** within last 14 days?

□ Yes	□ No

Have you experienced **repeated dry cough** within last 14 days?

□ Yes	□ No

Have you experienced **sore throat** within last 14 days?

□ Yes	□ No

Have you experienced **shortness of breath** within last 14 days?

□ Yes	□ No

Have you experienced **changes in sense of smell or taste** within last 14 days?

□ Yes	□ No

If you answer yes to one or more of the questions above, please contact your general physician or hotline 1450. **You cannot participate in the study.**

Have you been **tested positive for SARS-CoV-2** within last 14 days?

□ Yes	□ No

**If you answer yes to the last question, you cannot participate in the study.**

*Answered only if required by current Covid-19 guidelines:*

Have you been in a **corona risk area** within last 14 days?

□ Yes	□ No

Have you been in **quarantine** or ordered **“self-observation”** within last 14 days?

□ Yes	□ No

Do you have a valid **negative SARS-CoV-2 test result** that is not older than 72 hours (PCR test) or 48 hours (rapid antigen self-test)?

□ Yes	□ No

🡪 If ‘no’, please conduct a rapid antigen self-test at home.

-	-	-	-	-	-	-	-	-	-	-	-	-

**Current body temperature:**

At arrival	+30 min

Note, if body temperature is **37.8°C or higher** at arrival, the participant cannot participate in the study. Otherwise, please repeat the measurement 30 minutes later. If body temperature is **37.5°C or higher** at arrival, the body temperature needs to be 37°C or lower after 30 minutes for participation in the study.

**Special circumstances that may explain body temperature (e.g., cold/ hot environmental temperature, most recent physical activity such as exercising)?**

**Appendix C: Descriptives Home Setting and Preference for Study Location**

| **Item** | **Response** | **Frequency** |
| --- | --- | --- |
| **#1 Living condition** | Living in a shared apartment | 9 |
|  | Living alone | 4 |
|  | Living together with partner | 3 |
|  | Living with parents | 1 |
| **#2 Number of people living in** | 1 person | 4 |
| **participants’ apartments** | 2 persons | 6 |
|  | 3 persons | 4 |
|  | 4 persons | 3 |
| **#3 Room A** | Bedroom | 9 |
| **participant room** | Kitchen | 5 |
|  | Office | 1 |
|  | Bathroom | 1 |
|  | Living room | 1 |
| **#4 Room B** | Living/ dining room | 8 |
| **(placebo-)TSST room** | Bedroom | 7 |
|  | Office | 1 |
|  | Kitchen | 1 |

| **Would you have preferred that the study takes place at the university instead of your home? (German: „Hätten Sie die Studie lieber an der Universität durchgeführt anstatt bei Ihnen zu Hause?“)** | | |
| --- | --- | --- |
| **Response** | **Frequency** | **Reasons provided by participant** |
| „Yes, I would have preferred that the study takes place at the university“ (German: “Ja, mir wäre lieber gewesen, die Studie an der Universität durchzuführen”) | 5 | - Easier to distance yourself from the stress test (German: „An der Universität wäre es mir leichter gefallen, mich vom Stresstest abzugrenzen als in meinem eigenen Zimmer“)  - The invasion in your privacy is comparable with the slight feeling of helplessness (German: „Der Eingriff in die Privatsphäre ist mit einem leichten Gefühl der Hilflosigkeit zu vergleichen“)  - Additional stress factor: stress test in your own home; the silent TSST panel were perceived as „intruders/ burglars“ (German: „Zusätzlicher Stressfaktor, die unangenehme Situation in den eigenen vier Wänden zu erleben. Die beiden schweigsamen VL habe ich ein wenig wie ‚Einbrecher‘ (stark überzeichnet formuliert) wahrgenommen“) |
| 6x „No, I was glad that the study took place at my home“ (German: “Nein, ich war froh, sie zu Hause durchführen zu können”) | 6 | - interesting; new association with your home (another side, life) (German: „Es war interessant, so ein Experiment zu Hause durchzuführen. Bringt irgendwie Vielseitigkeit/ Leben in die Assoziation mit der eigenen Wohnung“)  - comfortable, all in all less time-consuming (German: „gemütlicher und insgesamt weniger Zeitaufwand“)  - no travel time (German: „keine Fahrzeit“)  - fast, no travel (German: „So ging es schnell und ich hatte keinen Stress mit der Anfahrt.“)  - less time-consuming (German: „weniger Zeitaufwand“) |
| „Neither nor“ (German: “Weder noch”) | 6 | - on the one hand comfortbale at home, on the other hand keep distance (German: „einerseits Ruhe von zuhause anderseits schön Distanz zu wahren“)  - would have taken it more serious at the university, felt artificial at home (still felt stressed); very interesting experience (German: „Ich wäre den Stresstest seriöser angegangen, hätte er an der Uni stattgefunden. Bei mir zuhause hat es dann doch sehr konstruiert gewirkt (gestresst war ich aber dennoch). Trotzdem eine sehr interessante Erfahrung“)  - less time-consuming at home; in general, irrelevant where study takes place (German: „zwar hat mir der Besuch zu Hause Zeit gespart, prinzipiell wäre es jedoch egal gewesen, wo die Studie durchgeführt worden wäre.“) |

**Appendix D. Additional statistical analyses and results**

**Appendix D.1: Illustration of stress responses using individual data points**

Figure C: Individual data points and averaged trajectories of stress responses: A) salivary cortisol, B) salivary alpha-amylase, C) VAS perceived stress, and D) heart rate.

Salivary samples and VAS perceived stress were obtained at the following timepoints: -10, -1, +1, +10, +20, +30, +45, and +60 min relative to the task. For heart rate, averaged 5-min intervals were calculated for baseline (at the end of the acclimation period, corresponding 10-15 min before task), speech preparation and anticipation (corresponding 5 min before speech), speech, arithmetic task, post task (0-5 min after task), recovery I (5-10 min after task), recovery II (20-25 min after task), recovery III (30-35 min after task), recovery IV (45-50 min after task), and recovery V (55-60 min after task).

**Appendix D.2: MELM statistics using non-transformed data**

The MELMs revealed significant time × condition interactions for cortisol: *F*(7, 239.01) = 12.89, *p* < 0.001; sAA: *F*(7, 238.99) = 3.49, *p* < 0.01; HR: *F*(9, 304) = 3.38, *p* < 0.001; VAS: *F*(7, 239.02) = 6.79, *p* < 0.001. Simple effects of condition for each time point were probed using estimated marginal means with Tukey-adjusted pairwise comparisons. Results revealed that, as predicted, the TSST induced stronger cortisol responses to stress than the placebo TSST, corresponding with all timepoints associated with stress reactivity and recovery (timepoints 3, 4, 5,6,7, 8; all *t*s > 2.44; *p*s ≤ 0.01). Likewise, for sAA, HR, and VAS, compared to the placebo, stress induction via the TSST was associated with stronger reactive increases, corresponding with all timepoints associated with stress reactivity: sAA (timepoint 3; *t* = 5.42; *p* ≤ 0.001); HR (timepoints 2, 3, 4, 5 ; all *t*s > 2.7; *p*s ≤ 0.001); VAS (timepoints 2, 3, 4; all *t*s > 3.63; *p*s <  0.001).

**Appendix D.3: Bayesian statistics**

We analyzed the effect of condition on stress reactive changes in biological (cortisol, sAA, HR) and psychological (VAS) stress markers using Bayesian linear mixed-effects models estimated via Markov Chain Monte Carlo sampling (four chains of 4,000 iterations each, with a warm-up of 1,000) (Gelman et al., 2013; Kruschke, 2014). Four separate models predicted the stress markers (cortisol, sAA, HR, and VAS) respectively, with log-transformed dependent variables z-scaled across all observations. Predictors included condition order (1 = 0, 2 = 1), sampling times (time 1 = 0, time 2 = 1, etc.), condition (placebo= 0, TSST = 1) as well as the time x condition interaction. The intercept of participants was added as a random effect. Priors were set to weakly informative distributions for all predictors. Specifically, we used a normal distribution with a mean of 0 and an SD of 2 for all predictors (Gill & Witko, 2013). All analyses were conducted in R (R Core Team (2025) version 4.5.1) using the brms package (Bürkner [& Others, 2017)](https://paperpile.com/c/4XezKy/bdr2j). When the 95% credible intervals for a parameter excluded zero, we interpreted this as strong evidence for the effect. We decomposed interaction effects using the emmeans package (Lenth, Piaskowski; 2025).

Result summary

To assess the effects of condition on stress reactive changes in biological (cortisol, sAA, HR) and psychological (VAS) stress markers, we fitted four Bayesian linear mixed models with each stress marker as the dependent variable and fixed effects of condition order, time, condition, time x condition interaction and a random intercept for ID. For all analyses, we defined the simple-effect contrast as Δ= Condition2 (placebo TSST) − Condition1 (TSST-home). Negative values are indicative of lower cortisol in placebo TSST than in the TSST-home. Credible differences are those where the 95% highest posterior density (HPD) credible interval (CrI) excludes 0. We report the results of these analyses for each stress marker below.

**Cortisol:**

The convergence for the model was satisfactory (all R̂ = 1.00). The random-intercept SD was 0.03 (95% CrI [0.00, 0.10]) and the residual SD was σ=0.61 (95% CrI [0.56, 0.66]). At baseline (i.e. time1) the conditions did not credibly differ, whereas at the time points associated with stress reactivity and recovery (time 3 to time 8), cortisol levels in the placebo TSST were credibly lower than the TSST-home. Posterior estimated marginal means and simple-effect contrasts (with 95% CIs) are reported for each time point:

Time 1: Δ  = 0.121, 95% CrI [-0.277, 0.504] (uncertain).

Time 2: Δ  = 0.272, 95% CrI [-0.149, 0.662] (uncertain).

**Time 3: Δ  = -0.551, 95% CrI [-0.959, -0.144] (credible; Condition2 < Condition1).**

**Time 4: Δ  = -1.572, 95% CrI [-1.987, -1.168] (credible; Condition2 < Condition1).**

**Time 5: Δ  = -1.877, 95% CrI [-2.273, -1.456] (credible; Condition2 < Condition1).**

**Time 6: Δ  = -1.716, 95% CrI [-2.126, -1.318] (credible; Condition2 < Condition1).**

**Time 7: Δ  = -1.644, 95% CrI [-2.056, -1.245] (credible; Condition2 < Condition1).**

**Time 8: Δ  = -1.467, 95% CrI [-1.856, -1.046] (credible; Condition2 < Condition1).**

**Amylase:**

The convergence for the model was satisfactory (all R̂ = 1.00). The random-intercept SD was 0.04 (95% CrI [0.00, 0.13]) and the residual SD was σ=0.76 (95% CrI [0.69, 0.83]). While at baseline (i.e. time1), the conditions did not credibly differ, at the time point associated with peak stress reactivity (time 3), amylase levels in the placebo TSST h were credibly lower than the TSST-home condition. Posterior estimated marginal means and simple-effect contrasts (with 95% CIs) are reported for each time:

Time 1: Δ  = 0.336, 95% CrI [-0.154, 0.789] (uncertain).

Time 2: Δ  = 0.050, 95% CrI [-0.446, 0.565] (uncertain).

**Time 3: Δ  = -1.419, 95% CrI [-1.936, -0.919] (credible; Condition2 < Condition1).**

Time 4: Δ  = -0.247, 95% CrI [-0.761, 0.253] (uncertain).

Time 5: Δ  = -0.414, 95% CrI [-0.898, 0.094] (uncertain).

Time 6: Δ  = -0.007, 95% CrI [-0.518, 0.514] (uncertain).

Time 7: Δ  = -0.031, 95% CrI [-0.528, 0.481] (uncertain).

Time 8: Δ  = -0.150, 95% CrI [-0.671, 0.347] (uncertain).

**Heart Rate:**

The convergence for the model was satisfactory (all R̂ = 1.00). The random-intercept SD was 0.03 (95% CrI [0.00, 0.08]) and the residual SD was σ=0.55 (95% CrI [0.51, 0.59]). At baseline (i.e. time 1), the conditions did not credibly differ, however at the time points associated with stress reactivity (time points 2, 3, 4, 5), heart rates in the placebo TSST were credibly lower than the TSST-home condition. Posterior estimated marginal means and simple-effect contrasts (with 95% CIs) are reported for each time:

Time 1: Δ = 0.119, 95% CrI [-0.230, 0.470] (uncertain).

**Time 2: Δ = -0.596, 95% CrI [-0.966, -0.236] (credible; Condition2 < Condition1).**

**Time 3: Δ = -0.804, 95% CrI [-1.189, -0.457] (credible; Condition2 < Condition1).**

**Time 4: Δ = -0.754, 95% CrI [-1.124, -0.396] (credible; Condition2 < Condition1).**

**Time 5: Δ = -0.529, 95% CrI [-0.890, -0.149] (credible; Condition2 < Condition1).**

Time 6: Δ = -0.260, 95% CrI [-0.630, 0.104] (uncertain).

Time 7: Δ = -0.237, 95% CrI [-0.585, 0.139] (uncertain).

Time 8: Δ = -0.119, 95% CrI [-0.491, 0.237] (uncertain).

Time 9: Δ = -0.099, 95% CrI [-0.468, 0.269] (uncertain).

Time 10: Δ = 0.040, 95% CrI [-0.326, 0.406] (uncertain).

**VAS**

The convergence for the model was satisfactory (all R̂ = 1.00). The random-intercept SD was 0.04 (95% CrI [0.00, 0.13]) and the residual SD was σ=0.76 (95% CrI [0.70, 0.83]). While at baseline (i.e. time1) the conditions did not credibly differ, time points associated with stress reactivity (time points 2, 3, 4, 5), showed credibly lower subjective stress levels in the placebo than the TSST-home condition. Posterior estimated marginal means and simple-effect contrasts (with 95% CIs) are reported for each time:

Time 1: Δ  = -0.037, 95% CrI [-0.495, 0.456] (uncertain).

**Time 2: Δ  = -0.865, 95% CrI [-1.380, -0.356] (credible; Condition2 < Condition1).**

**Time 3: Δ  = -1.637, 95% CrI [-2.142, -1.147] (credible; Condition2 < Condition1).**

**Time 4: Δ  = -0.811, 95% CrI [-1.312, -0.303] (credible; Condition2 < Condition1).**

**Time 5: Δ  = -0.576, 95% CrI [-1.080, -0.083] (credible; Condition2 < Condition1).**

Time 6: Δ  = -0.278, 95% CrI [-0.775, 0.217] (uncertain).

Time 7: Δ  = -0.238, 95% CrI [-0.747, 0.263] (uncertain).

Time 8: Δ  = -0.278, 95% CrI [-0.805, 0.225] (uncertain).

**Appendix E: Brief comparison between TSST versions**

|  | Laboratory TSST | Virtual-reality (VR) TSST | Online-based TSST | Home-based TSST |
| --- | --- | --- | --- | --- |
| Personnel resources | 1 experimenter  2 members TSST panel (originally 3 members) | 1 experimenter | 1 experimenter  2 members TSST panel (unless pre-recorded videoconferences are used) | 1 experimenter  2 members TSST panel |
| Location | Research institute | Research institute | Participants’ home (if applicable, experimenter onsite) and research institute (TSST panel online, experimenter online) | Participants’ home |
| Travel burden | Participants | Participants | Participants (if applicable, experimenter) | Research team |
| TSST-committee | In-person, physical presence | Virtual (avatar, pre-recording or live streaming) | Virtual (pre-recording, live streaming) | In-person, physical presence |
| Degree of immersion/ intrusion | High: physical presence; anticipatory stress due to laboratory setting | Moderate to high: depending on VR equipment; anticipatory stress due to laboratory setting | Moderate: virtual | High: physical presence; intrusion to participants’ private space (home setting) |
| Spatial requirement | 2 rooms (including tables, chairs) | 2 rooms (including tables, chairs) | 1 room (table, chair) | 2 rooms (including tables, chairs) |
| Technical equipment | Video camera, microphone | VR equipment | PC/ notebook with camera, microphone and stable internet connection | Video camera, microphone |
| Ecological validity | Low (laboratory setting) | Low (laboratory setting) | High (at participants’ natural environment, home setting) | High (at participants’ natural environment, home setting) |
| Standardization of TSST protocol | Semi-standardized;  adaptive to participants’ behavior | Semi-standardized;  if live streaming, adaptive to participants’ behavior  if pre-recording or avatar standardized panel | Semi-standardized;  if live streaming, adaptive to participants’ behavior | Semi-standardized;  adaptive to participants’ behavior |
| Target populations | Adults | Adults | Adults (access to internet is required) | Adults; including hard-to-reach, vulnerable populations |
| Application | Laboratory-based research | Laboratory-based research | Field studies | Epidemiological studies, field studies |

In summary, the required personnel and material resources are comparable among the TSST version, with minor differences depending on the required equipment for the set-up (e.g., VR equipment, computer including internet). For the TSST-home, the travelling burden is shifted from participants to the research team, resulting in additional travel time and transportation of the study material. The overall advantage of the TSST-home is that it allows us to reach and include participants who would otherwise decline to travel to a research institute for a study for individual reasons. For example, representative epidemiological studies might be (self-)selective-biased if only individuals living near research institutes participate, while individuals living further away, for example in the countryside, might be under-represented because they do not want to or cannot easily travel to the research institute.
